# General practitioners’ perspectives on a proposed new model of service delivery for primary care management of knee osteoarthritis: a qualitative study

**DOI:** 10.1186/s12875-017-0656-7

**Published:** 2017-09-07

**Authors:** Thorlene Egerton, Rachel Nelligan, Jenny Setchell, Lou Atkins, Kim L. Bennell

**Affiliations:** 1 Centre for Health, Exercise and Sports Medicine, The University of Melbourne, Melbourne, Australia; 2 School of Health and Rehabilitation Sciences, The University of Queensland, Brisbane, Australia; 3 University College London, London, UK

**Keywords:** Osteoarthritis, Primary care, Models of care, Implementation, Qualitative, General practitioner

## Abstract

**Background:**

Effective management of people with knee osteoarthritis (OA) requires development of new models of care, and successful implementation relies on engagement of general practitioners (GPs). This study used a qualitative methodology to identify potential factors influencing GPs’ engagement with a proposed new model of service delivery to provide evidence-based care for patients with knee OA and achieve better patient outcomes.

**Methods:**

Semi-structured telephone interviews with 11 GPs were conducted. Based on a theoretical model of behaviour, interview questions were designed to elicit perspectives on a remotely-delivered (telephone-based) service to support behaviour change and self-management for patients with knee OA, with a focus on exercise and weight loss. Transcripts were analysed using an inductive thematic approach, and GPs’ opinions were organised using the APEASE (affordability, practicability, effectiveness, acceptability, safety/side effects and equity) criteria as themes.

**Results:**

GPs expressed concerns about potential for confusion, incongruence of information and advice, disconnect with other schemes and initiatives, loss of control of patient care, lack of belief in the need and benefits of proposed service, resistance to change because of lack of familiarity with the procedures and the service, and reluctance to trust in the skills and abilities of the health professionals providing the care support. GPs also recognised the potential benefits of the extra support for patients, and improved access for remote patients to clinicians with specialist knowledge.

**Conclusion:**

The findings can be used to optimise implementation and engagement with a remotely-delivered ‘care support team’ model by GPs.

**Electronic supplementary material:**

The online version of this article (doi: 10.1186/s12875-017-0656-7) contains supplementary material, which is available to authorized users.

## Background

Osteoarthritis (OA) is a major cause of pain and disability, affecting 3.8% of the global population [1]. It leads to a considerable global burden on health services [2]; for example, it is the 6th most managed problem by general practitioners (GPs) in Australia [3]. The knee is one of the most commonly affected joints and pain from knee OA can lead to severe loss of function if the disease progresses [4]. With the forecast increase in prevalence [1], the already considerable health expenditure and burden to society of this condition will continue to rise [5].

Clinical practice guidelines for knee OA emphasise non-surgical, non-drug treatment, in particular, education, self-management support, exercise and weight loss, as core OA management [6–8]. In most countries, people with knee OA are managed predominantly in primary care by their GP or family doctor [9]. Optimal care requires patients to be empowered to self-manage with lifestyle interventions that require long-term behavioural change, which creates additional management challenges for GPs [10, 11]. Unsurprisingly, current management of the condition can be variable and is often inconsistent with recommended practice [3, 11, 12]. Studies from many parts of the world reveal over-reliance on imaging and drugs compared with self-management and lifestyle options [13], and referrals to orthopaedic surgeons can be higher than referrals for exercise [13]. This may explain in part why, despite the range of effective interventions available, 68% of people with arthritis in Australia stated they were “doing badly” or “fairly badly” regarding how their lives were affected by the condition [14].

Previous studies carried out in Australia [10, 15] and other countries [16–21], have highlighted barriers to recommended knee OA management. These include: time constraints; lack of skills and confidence to facilitate shared decision-making and support patient self-management; cost and geographical barriers to patients accessing exercise, weight-loss and psychological help; inaccurate beliefs about the disease process and progression among health professionals and patients; and professional biases including defaulting to treatments that are easiest to deliver, general negativity towards managing the condition, and ‘normalisation’ instead of validation of symptoms [10, 19, 20, 22, 23]. Many of these barriers to best practice may be exacerbated for people with multiple morbidities, mobility limitations, language barriers, and/or those living in geographically remote areas [24]. There have been many calls for new models of care to address the failures in delivery of optimal care to all people with knee OA [25]. A model incorporating a dedicated team to support GPs to manage patients with knee OA and assist patients with long term self-management and behaviour change may address these recognised barriers.

**Fig. 1:**
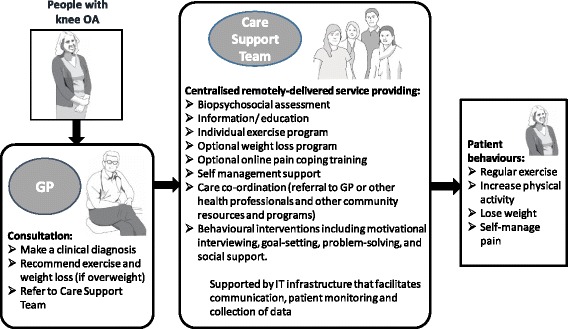
Schematic representation of the proposed model of service delivery for primary care management of knee OA including the ‘Care Support Team’

We therefore designed a new model for Australian primary care management of knee OA (Fig. 1), which was underpinned by theory [26–28] and informed by evidence [10, 29–31]. The model was developed by a multi-site, multi-disciplinary group including general practice researcher/clinicians, with broad stakeholder input via several online surveys, meetings and a focus group which included consumers and general practitioners. The model addresses many of the identified barriers to recommended practice and incorporates evidence-based components of chronic disease models of care [13, 32]. Our model includes a multi-disciplinary team of health professionals using remote-delivery options (primarily telephone) to provide ongoing ‘care support’. Remotely-delivered models of care are effective and can improve access on a population-level by reducing cultural, language, socioeconomic and geographical barriers [33–35]. In our model, the GP refers the patient to the ‘care support team’ following a brief initial consultation. The ‘care support team’ staff will have skills in health behaviour change plus expertise in current best practice for knee OA management.

The use of remotely-delivered services is increasing, particularly for monitoring and/or management of chronic health problems. There is a corresponding increase in research investigating effectiveness and exploring the acceptability and integration of such models [36, 37]. Most exploratory work has focussed on patient perspectives, with a small body of literature considering the views of the primary health care staff the new service impacts. Other models to manage long-term conditions such as heart disease and diabetes have been met with ambivalence, scepticism about effectiveness and/or concerns about fragmentation of care [36]. The importance of considering the impacts on existing inter-professional relationships has been highlighted [37] as well as the need to facilitate staff acceptance as part of implementation [38]. In particular, the potential for GPs to feel that new services undermine their authority and autonomy must be understood and sensitively handled [39]. These previous findings highlight the importance of pre-empting conflicts or resistance by exploring perspectives before implementation.

Since GPs need to refer patients to the ‘care support team’ in our proposed model, successful implementation will rely on GP acceptance and engagement. Therefore, to support implementation, we carried out a qualitative study to identify GPs’ perspectives on potential barriers and facilitators to engagement with the proposed model to support knee OA management. The methods and findings will be of interest to those developing similar remotely-delivered services for chronic pain or musculoskeletal disease management.

## Methods

This qualitative study is nested within a larger project to develop, implement and evaluate a new model of service delivery for the primary management of knee OA. This study was carried out after the initial model design and before the implementation plan was developed. The Melbourne University School of Health Sciences Human Ethics Advisory Group approved the study (ID 1545504) and all participants gave written and verbal informed consent.

### Study design

Data were collected by semi-structured telephone interviews and analysed using interpretive thematic analysis techniques directed towards identifying barriers and facilitators to GP engagement with the model. Telephone interviews were chosen for their convenience due to GPs’ high workload and geographical spread. Interviews had two sections: the first explored GPs views on diagnosing OA and delivering exercise and weight loss interventions within the current service model. This section is not reported in this paper. The second section, reported here, explored GPs’ perceptions of the proposed new model. The COREQ-checklist was used to ensure study rigour, trustworthiness and transparent reporting [40]. The study satisfied the relevant COREQ criteria with details provided Additional File 1: COREQ checklist.

### Participants

The proposed new model of service delivery was designed to be implementable in the Australian primary health care system, which includes GPs working in large and small practices in both metropolitan and rural/regional settings. Practices are mostly privately owned and run as small businesses. We purposively sampled GPs from metropolitan, regional, large (≥4 GPs) and small (<4GPs) practices with a mix of GP sexes, ages and years of experience. Sample size was initially set to be a minimum of 10 with the final size guided by data saturation [41]. Inclusion criteria were: GP qualification, current primary care practice and at least one patient with knee OA per month. GPs registered with the Victorian Primary Care Research Network or who were personal contacts of the researchers were initially invited, with snowballing used for ongoing recruitment. Potential participants were emailed study information and a consent form. Participants were asked to pre-read information summarising the proposed model. The pre-reading information provided via email consisted of three pages explaining the rationale for setting up a new service, the key features and functions of the new service and the purpose and focus of the study (10–15 min reading). The information sheet was intended only for use in this qualitative study and was provided to save time during the interviews. The interviewer checked the material had been read as part of the introductory preamble. All GPs reported having read the information, and the interviewer provided reminders or clarification as necessary during the interviews. Demographic details collected included: age, sex, years of practice, location (metropolitan/regional), practice size, other staff in the practice and frequency of patients consulting for knee OA.

### Data collection

A trained qualitative researcher [RN] conducted interviews. The interviewer was a female physiotherapist who had no prior relationship with the participants or involvement in the new model design. All interviews were audio recorded, transcribed by a professional service and then checked for accuracy by the interviewer. Field notes included difficulties encountered during the interviews and the interviewer’s impressions of participants’ reactions to the study. Transcripts were anonymised using numbers representing the chronological order of interviews (GP1-GP11), and securely stored in line with institutional regulations. Participants were advised that the whole interview could be completed in 30 min but were not discouraged from extending the interview duration if willing. The interviews ranged from 30 to 90 min in length with most lasting about an hour. The interview questions for this study constituted roughly the second half of the interview although this data should not be considered entirely in isolation from the preceding conversation. Participants were offered a $50 shopping voucher for participation.

### Interview guide

The interview guide was semi-structured to prompt consideration of potential barriers and facilitators, whilst allowing flexibility for participants to raise issues and contribute their own ideas. The interview questions were informed by the COM-B (Capability/Opportunity/Motivation-Behaviour) theoretical framework [28] and developed in collaboration with a qualitative research expert [JS] and all members of the research team. The interview guide is provided in Table 1.

**Table 1:** Interview guide

Key topic	Questions
GP feelings about the proposed support service for patients with knee OA	Do you think the proposed service where management of knee OA patients is shared by GPs and the support team sounds like a good idea? How would you feel about referring your patients to such a service? Would you have any concerns about referring any of your patients to such a service? Do you think GPs would feel motivated to refer if such a service was available to them? What would increase or decrease their motivation? What do you perceive would be the benefits for patients, if any, of referring to the proposed service? For GPs? Would referral be compatible with how GPs feel about their role in managing knee OA? How do you feel it would impact on patient outcomes? How would referral to a ‘care support team’ impact on your own day-to-day practice and business? Do you have any other comments on the proposed support service?

### Data analysis

The analysis team consisted of two researchers [TE – senior researcher and qualified physiotherapist, and RN – experienced qualitative researcher and qualified physiotherapist] and a qualitative research expert to oversee the analytic process [JS]. Analysis was initially performed within an inductive thematic approach as described by Braun & Clark [42], which was anchored to the research question of identifying perceived barriers, facilitators or beliefs that could affect engagement with the proposed service. Findings were then organised into the APEASE (affordability, practicability, effectiveness, acceptability, side-effects/safety and equity) framework. This framework is an interpretative conceptual model developed to assist with the design of new interventions and services and to help evaluate intervention ideas [28] (Table 1 p23), and was used in this study to assist with organising and understanding our data. An iterative stepped approach to data analysis was used. First, TE and RN read transcripts as they became available and independently generated a list of potential codes and categories, organised manually using data management software (Microsoft Excel). Results were then discussed until they agreed on a provisional coding framework within the APEASE themes. After seven interviews, the data were reviewed in relation to the research question and minor modifications to the wording of some interview questions were made to probe deeper into relevant topics. Following the modifications, another four GPs were interviewed. These data were coded and since no new topics were identified we considered sufficient data saturation was achieved [41]. The co-authors [JS, LA, and KB] reviewed the data and coding and confirmed that the analysis was credible and the themes grounded in the data. An external reviewer (a GP not involved in the study) also reviewed the findings for credibility.

## Results

### Sample

From November 2015 through February 2016, we conducted 11 GP interviews. The GPs had a mix of sexes (64% women) and metropolitan versus regional practices (55% metropolitan), and a range of ages (34–67 years, mean 50.8), experience (5–44 years, mean 21.6) and practice size (1–24 GPs, mean 7.5). They self-reported seeing 1–40 (mean 12) knee OA patients per month, demonstrating sample heterogeneity.

Between one and six codes were identified within each of the themes (APEASE criteria). These codes are summarised in Table 2 and described in the following sections, with anonymised examples of supporting data. Additional participant quotations supporting each theme are provided in Additional File 2.

**Table 2:** Identified codes organised into the APEASE framework

Themes: APEASE criteria	Codes
1. Affordability	Influence of cost to patients
2. Practicability	Efficiency of referral procedure
	Efficiency and effectiveness of ongoing communication
	Fitting in with existing initiatives
3. Effectiveness	Is there a need?
	Will it improve outcomes?
4. Acceptability	Trust
	Personal relationships
	The burden of care on GPs
5. Safety/side effects	Worsening of outcomes
6. Equity	Patient diversity

#### Theme 1: Affordability

One code was identified in this theme: ‘*Influence of cost to patients’*. GPs expressed concern that uptake would be negatively impacted if patients were required to pay. For example: “*If there’s a cost [to patients], that could be a problem.” (GP2).* GPs generally felt that it should be funded by sources other than patients: “*Ideally it should be …provided for free.” (GP3).* One GP also said that GPs would be more likely to engage with the ‘care support team’ if it enabled “…*more affordable, accessible allied health.*” (GP3) for their patients. These data indicate that affordability to patients is a consideration for GPs who appeared more concerned on behalf of their patients than about any cost to themselves.

#### Theme 2: Practicability

GPs raised three main issues related to the practicalities of their engagement with the new service.

##### Efficiency of referral procedure

Many GPs emphasised the need to ensure referral procedures are streamlined in order to minimise impact on their busy schedules. For example, one said: “*It depends how simple [the referral] process is. I mean, if you’ve got a referral template that you’re simply filling in a few details, that doesn’t take a lot of time.” (GP7).* Most GPs also discussed the importance of avoiding duplication of assessment, minimising burden for patients, and avoiding unnecessary work for the care support providers.

*It’s how much information the team needs, how much of it is not necessary, will they repeat that themselves by way of that initial assessment. I think it’s important not to create more unnecessary work. (GP7)*.

##### Efficiency and effectiveness of ongoing communication

A second practical issue raised by almost all GPs was the quality and quantity of communication between the GP and the ‘care support team’, with one GP explaining: “*Communication would be important to us, so we’re kept in the loop.” (GP10).* GPs identified the need for effective, useful and timely channels of communication.

*I think it comes down to the practicalities to be honest for a lot of these systems whether they succeed or fail, and that’s about taking time with the communication that was set up and getting the foundation in place to be effective (GP1)*.

The majority of GPs’ concerns related to whether the ‘care support team’ would send information to the GPs. In particular, GPs wanted to be updated on the advice given and the plan made so they know what has been said to their patient.

*So they come back to the see the GP, the GP is not really sure what’s going on or where they’ve been or come with a request for such and such from this team and doesn’t really know why or what or whether it’s appropriate (GP6)*.

*We’re not having to spend a lot of time asking “Well, did you see this person? What did they do, what did they say?” (GP10)*.

##### Fitting in with existing initiatives

Thirdly, GPs raised the issue of whether the new service would be compatible or complementary with existing primary care initiatives for management of chronic disease, expressing concern that management could become complicated and confusing: *“How will this ‘care support team’ get a sense of that [holistic view] and link it with a chronic disease management plan? It could get quite messy…” (GP3).* The GPs also suggested there needs to be clarity about how the new service would integrate with existing schemes and payment structures and that an overcomplicated system may lead to inequity of care for their patients and/or reduce their uptake of the service.

*There’s all these other things that are happening in the background that will influence how GPs engage with a programme like this. Thinking about how this will fit into the regular work of a GP will make a big difference, to whether it succeeds or fails (GP3)*.

#### Theme 3: Effectiveness

Two codes were identified relating to the perceived effectiveness of the proposed new model.

##### Is there a need?

A range of views was expressed about the value of the service from not seeing any need at all, through to a belief that extra care for knee OA patients is vital. Some GPs felt there were already adequate skills and resources, either within their own skill set or within their practice staff, to support OA patient self-management and lifestyle change: *“I suppose, again my initial feeling is well, I’ve done the job for 30 years and one way or the other most patients seem to have done okay with something like OA.” (GP6).* The same GP also raised a concern about providing this service for a condition perceived as low priority: “*there’s a ton of other patients that I’d love to have a ‘care support team’ around... osteoarthritis doesn’t jump to the top of my tree of clinical issues and problems that I find difficult to manage” (GP6)*.

In contrast, many GPs indicated that help *was* needed, seeing benefit that their advice was “*going to be reinforced”* (GP5), that the service may *“integrate care”* (GP6), and could provide much needed extra *“encouragement”* (GP3). This positivity was exemplified by one GP: “*Yeah, so I think that it will be like a one stop referral shop, it will be fantastic as a team behind the patient, and that could be good.” (GP2)*. The positive comments regarding perceived need seemed to stem from a belief that for this type of condition, the advice and recommendations (such as lifestyle changes) may need to be reinforced or perhaps provided over several health care episodes. It was not necessarily that GPs saw the value of the service in providing something different or additional to their own care, but that more of the same would benefit patients.

There were thus mixed feelings amongst the GPs about the need for this service. Some GPs expressed views both for and against a need, indicating they held uncertain feelings about this issue. For example, GP6 (as quoted above) initially suggested the service was not needed and later highlighted the need for integrated care which he felt the service might support. It is possible that the perception of need may vary depending on the individual patient (as discussed under “patient diversity” below).

##### Will it improve outcomes?

GPs also expressed mixed views about whether the proposed service would lead to better patient outcomes. Some suggested outcomes would not improve because they believed advice already given at their practice would be unhelpfully repeated, that remote (telephone) delivery is not as good as face-to-face particularly in relation to exercise advice, and that advice to exercise and lose weight does not work.

*And what I found [from a similar service] was the patients didn’t mind the phone call - they loved it - but they actually didn’t lose weight. (GP8)*.

*I’m not sure how effective patients would find speaking to someone about exercise or … as opposed to face-to-face and having that personal contact (GP6)*.

To the contrary, some GPs believed the extra time and encouragement for the patient would result in better outcomes.

*All of that extra contact should, you would hypothesise, flow into more motivation and more actual change (GP11)*.

*Instead of writing Voltaren and saying use that now and again when it’s bad, and take paracetamol, and let’s get some exercises, and let’s lose some weight and these are all the things we need to look at, but here’s somebody who’s actually going to monitor and encourage and be part of all this journey with you (GP1)*.

One GP identified a further potential benefit of increased access to OA specialists: “*I guess if it’s much more specific advice of more nuance on parts of exercise and weight loss that I as a GP don’t know about well maybe that will help.” (GP3)*.

#### Theme 4: Acceptability

GPs raised several issues relating to the acceptability of the service, the most important being trust, but there was also consideration of the personal relationships with their health care team and the advantages to themselves.

##### Trust

The GPs raised several issues that appeared to be related to trust. For example, many GPs comments indicated hesitancy to embrace an *unfamiliar* new service. In particular, GPs commented on the importance of clearly understanding the roles and functions of the service, the ‘care support team’ members, and themselves within the model: “*We’d have to be clear about who is responsible - you know what the role of a GP is in this process and what the role of the ‘care support team’ is in the process as well.” (GP10)*. A few GPs raised concern regarding long-term service *sustainability* and discussed the importance of broad acceptance of the new service by patients, doctors and health service funders if it is to continue long term: “Y*ou sometimes get this kind of pessimism from GPs. It’s not that they don’t want better interventions, it’s just that they’re sceptical that they will truly become a routine easily accessible part of practice.” (GP3)*. These ideas seemed to relate to whether they trusted their efforts to embrace change would be worthwhile.

Another aspect of trust that was commonly raised by GPs was the importance of having *confidence* in the staff of a new service to deliver on promises, and further, that having this confidence would facilitate their promotion of the service to their patients.

*What you would need to know is that they’re competent to do what’s being asked of them, that they have access to resources and that they know when to refer back. The checks and balances. Because I would not want to think that they would be doing anything that would be exacerbating the problem or creating other difficulties, such as soft-tissue injuries, because of inappropriate management (GP7)*.

Some GPs also pointed out that interpersonal skills, such as developing rapport with patients, are vitally important. For example: *“Getting the right person providing the care support… a person who’s actually got the right communication skills and right insight and right expertise.” (GP3)*. These GPs seemed to indicate that they would like to be assured that staff would have these skills and reflected that their trust would be developed if patient feedback and outcomes were positive. For example: *“So that you can actually speak of [the service] as something you know rather than just something you’ve been told this is for OA and here’s your referral”. (GP1)*.

A final trust-related issue was that some GPs had concerns about *security of patient data* and information confidentiality during the referral process. They said that they would be hesitant to refer if they had doubts about who could access their patients’ information: “*Allowing patient confidentiality to go up in the Cloud and having external people looking at the confidential information; that really concerns me”. (GP8).* In short, trust, relating to familiarity, confidence in the credibility of the ‘care support team’ staff, sustainability and security of data, were all important to GPs. Interestingly GPs’ comments indicated a tendency to default to having doubt rather than having trust suggesting GPs don’t easily trust a new service.

##### Personal relationships

Overwhelmingly, the GPs expressed a belief that having a personal relationship with the people providing the service would be preferable if they are to feel comfortable referring their patients. As noted in this comment: *“The idea of handing a patient over to an anonymous group of people… I don’t see a great attraction”. (GP6)*. GPs expressed a desire to work closely with service staff, know their names, and be able to call and discuss a patient: “*If the ‘care support team’ were people that we’d actually met … That they become names to us, you know, rather than just anonymous.” (GP1).* GPs expressed concern that their job satisfaction may be diminished if they felt they were handing over the care of their patients to a third party with no further involvement: “*As the GP, how do I keep a sense of being involved in an ongoing situation? Am I just handing over to, a group of other people and I wash my hands of them?” (GP6)*.

##### The burden of care on GPs

Some GPs found the idea of having some of the burden of managing this patient group taken away appealing. The reasons given were mostly about the time it currently takes to refer to multiple allied health services: *“And that would be helpful for the GP instead of sort of trying to figure out ‘Where can I send these people?’ The ‘care support team’ could take care of that.” (GP10)*. There was also some appeal for a lessening of their own ‘responsibility’ in terms of managing this condition: “*If what’s being proposed actually takes some of the load away from frontline GPs that might well be all the incentive that you need.” (GP7)*.

#### Theme 5: Side effects/safety

The GPs discussed how the service could lead to ‘*Worsening of outcomes’*. The addition of a ‘care support team’ may add complexities to management, increase paperwork, and/or lead to them feeling disconnected with their patient’s care. Some GPs also foresaw potential for confusion about the treatment plan.

GPs also raised the potential for issues resulting from incongruence of patient advice and information. They felt that differences in the advice given by the ‘care support team’ with their own explanations could lead to a need for them to spend extra time dealing with the conflicting messages.

*They’ll pick up a whole lot of problems, which create more problems for you and the patient, which weren’t ever there to start with… (GP2)*.

*There’s a possibility that … the way that they approach the problem is going to be a little bit different to mine… every now and then it’s some seemingly innocent or innocuous comment the patient turns over and then brings it back to you and you have to sort of spend time addressing that (GP5)*.

#### Theme 6. Equity

The theme of Equity is concerned with whether an intervention reaches all the intended recipients or whether it will disadvantage some groups. In this theme, GPs discussed the service’s ability to manage the diversity among patients with this problem and also the service’s ability to provide better access and support for rural patients.

##### Patient diversity

Some GPs were concerned the service would not be able to provide individualised management for a very diverse population. There were concerns that staff would just be “following a script” (GP3): *“So I’m thinking that a one size fits all [service] that will work because someone has osteoarthritis of the knee is a little bit of a pipedream” (GP3)*. Hearing and cognitive difficulties were raised as barriers for some patients to being able to interact with the service. GPs also had beliefs about the level of disease severity that could be effectively managed – that is, whether people with very mild or very severe joint disease would benefit. “*Some patients might have very mild arthritis and they won't want to [access the service]. Some patients are just waiting for their new knees, and they won't be relevant…” (GP2)*. The inability of a remote service to provide locally relevant information was also seen as a barrier to individualised care. Thus there were several concerns raised about the ability of the service to meet diverse individual needs.

GPs also suggested there would be variations in patient engagement with the service. Several GPs had a degree of scepticism about whether many patients would embrace such a model, particularly because of the remote-delivery aspect. For example, one GP said: *“I think it’s just sort of like those cold calls, … what gives the validity that this person can help me?” (GP1)*. However, another suggested that this issue might be more generic: “*There is going to be a group both of patients and GPs who just don’t want to engage with that type of model. But I think that will be the case no matter what model is designed or developed.” (GP5)* These comments indicate that GPs were concerned that the new model of service would only be adopted by certain individuals.

Two GPs said they believed the service could increase access to support for rural patients*,* and identified this as a major potential benefit: “*I think accessibility is a huge pro, so if it’s remote then it can be accessed by phone or internet or something whenever the patient is free.” (GP9)*. The potential for remotely-delivered services to improve access to care for some people with knee OA was considered an important strength of the proposal during initial service design. That few GPs noted improved access for rural patients, non-English speakers or those with mobility limitations as a potential positive is a finding in itself as it may suggest that equity is not of particular importance to the GPs in this cohort or something that they feel they normally have little influence on.

## Discussion

This study used qualitative methodology to understand the factors that could influence GPs’ engagement with a proposed new service delivery model to support care for patients with knee OA, help close the evidence-practice gap, and improve patient outcomes. Our participating GPs raised issues related mainly to the perceived effectiveness, perceived need, acceptability and practicability of the potential service.

Many of the GPs expressed concerns the service would be ineffective. It is possible that their concerns are realistic; however some of these negative views may reflect inaccurate or inadequate knowledge of the evidence for effectiveness of treatment options for OA. Previous qualitative studies have shown that GPs’ knowledge of current guideline recommendations can be lacking and that some doubt the effectiveness of lifestyle interventions [19, 21]. Such doubts were found to be a predictor of non-referral by GPs of OA patients to self-management programs in another Australian study [43]. Further, in a UK study of motivations to continue an intervention (with many similarities to our proposed model), perceived effectiveness was found to influence continuation behaviour [44]. Perceived effectiveness also concerns the ability of the *model* to achieve the predicted benefits. One study showed that practitioners want proof [45]. Concurring with our findings, in addition to empirical proof, perceived effectiveness is also determined by feedback from patients [44]. Our study findings thus highlight, similar to other literature, that for a new service to be taken up by referring GPs, they would have to know that the service is both supported by empirical evidence and accepted by patients. This has implications for implementation of such a service.

There were mixed perceptions about there being a *need* for such a service. Our findings support other research that has identified GPs as less likely to refer to self-management programs if they believe the patient can be adequately managed with the resources available to their practice [43]. These researchers suggested this belief reflects a lack of understanding about the differences between self-management programs and other practice nurse, allied health and community services [43]. In addition, GPs may perceive the needs of the patient differently to their actual needs. In a study on patient perspectives of OA management, patients felt GPs focussed more on the pathology and treatment of disease and less on their main concerns (e.g., pain and fear of disability) [21]. A lack of perceived need for the service also demonstrates lack of awareness that the health care system is currently failing to deliver optimal care to many patients with knee OA [3, 46, 47], and that evidence supports a need for new models of service delivery [48]. These gaps in understanding would need to be addressed to ensure positive uptake of a new service by GPs.

Lack of trust was identified as another major potential barrier to uptake. Trust issues were related to familiarity, credibility and perceived sustainability. Familiarity with the scope of functions provided by the service, roles of the GPs and ‘care support team’ staff within the service, and the mechanisms of referral and communication were all areas of concern for the GPs and may contribute to change resistance. In addition, having a personal relationship with the ‘care support team’ was seen as important for facilitating communication and would likely also have an important role in developing trust. In a chicken and egg scenario, GPs indicated they prefer to refer to people they have a personal relationship with and who have gained their trust, yet they need to use the service in order to build familiarity and these connections. This finding suggests that direct personal engagement with GPs would be a mechanism towards uptaking of any new service.

Change that challenges existing practice is not always welcomed [45]. The introduction of a telehealth program in Australia lead to resistance from GPs due to concerns about the other health practitioners encroaching on their area, the possible conflict with the business model of GP practice, and importantly, because GPs are too busy and too tired of changes to embrace another new initiative [45]. Similar views were revealed in our findings with the concerns raised about losing control of their patients’ care and the potential for the care provided by the remote team to be inconsistent with their own management. A few of the GPs also commented that the likelihood of the change being sustained would influence their engagement, possibly reflecting a degree of ‘change fatigue’ [49]. GPs spend long periods of their careers working in an environment where their individual professional autonomy is paramount, which is at odds with a system that tries to impose changes and constraints on them [50]. Autonomy and maintaining a level of control of patient care, important for GP job satisfaction, need to be supported during service changes.

Most GPs appeared to assume that the service would not be able to cater for the heterogeneity of the OA patient population they encounter. Similar findings were reported by Pitt et al. [43] regarding referral of OA patients to self-management programs. Their GPs believed that patients would either fail to attend, fail to change their behaviour, feel as if they did not fit in, or become anxious in response to the information promoting self-management. Clinician acceptance of a new service has been shown to be the most important driver of change in other primary care initiatives [45, 51] and is vital for sustainability [52]. Ensuring the changes required of GPs are minimal should help tip the balance in favour of change being accepted rather than resisted [53].

A number of GPs in our study recognised a need for additional help to manage this group of patients. Some also found the reduction in the burden of care for this group appealing and some suggested that the service could result in improved access to help for patients, particularly those in rural locations. Other studies have shown that managing chronic diseases within an acute episodic care model creates difficulties for GPs [22, 54]. GPs generally have negative attitudes towards managing knee OA and reported the psychological burden of managing a condition that they believe they have little potential to positively impact [19–22]. For this reason, it is perhaps surprising that comments in favour of the service were relatively few in our study. Following the trial in the UK of a service with many similar components to the service proposed here, GPs who experienced having the additional support for patients reported finding it easier to manage consultations with this group who they had previously thought of as having a ‘difficult’ condition [44]. Thus GPs in our study may not have anticipated ways in which the service may directly benefit them.

The results of this study provide insight into how engagement with the proposed new service might be facilitated. In most cases the concerns raised by GPs seemed legitimate and all warranted consideration when further developing our service. In particular, the issue of trust, which indicated GPs’ motivation to avoid exposing their patients to potential negative impacts, warrants careful attention when new services are introduced. Practical solutions to concerns about referral, communication and data security, ideally built into existing practice software, may help alleviate some resistance. Proactively creating opportunities to interact directly with personnel at an early stage would help build familiarity and relationships. Positive feedback from patients and unanticipated direct benefits to themselves may also help with longer term acceptability. Education to build GPs’ knowledge and confidence about recommended primary care management of people with knee OA and the gaps in current care should be provided. Marketing of the service should highlight how it will cater to the diverse needs of the heterogeneous population and provide referral guidance. Marketing strategies should also facilitate familiarisation with the staff and their functions, and explain how the service will fit in with other local chronic condition management initiatives and GP reimbursement procedures.

A number of contextual factors may have influenced the results of this study. The geographical locations of the participants (one state in Australia) may affect the transferability of the findings to other locations and this would need to be considered if results are applied to other settings. However, representation from regional as well as metropolitan practices likely added to the breadth of findings. The nature of qualitative research means the interviewer’s perspective and the analysers’ opinions influence the findings [55]. The analysis team were predominantly physiotherapists but the inclusion of feedback from a psychologist and a practicing GP would have helped provide a balanced perspective. The relatively limited quantity of data may have reduced the depth of understanding possible, however the use of the pre-reading will have optimised the use of the available interview time. Our method of combining inductive and deductive approaches warrants attention. The initial inductive analysis allowed unconstrained interpretation of the data at first. The later fitting of the data to the APEASE framework posed some difficulties whereby some data could be fitted to more than one APEASE theme and some themes had limited data making it difficult to fully understand GPs perspective on some of the APEASE criteria (eg. Affordability). However, the ‘analyst-driven’ approach [42] of using the APEASE framework had the advantage of ensuring we were comprehensive and considered many aspects important for new intervention development. We also found the framework worked well to subsequently help translate findings to strategies for facilitating engagement with a new service. By its qualitative design this research cannot know what GPs will actually do in the event of the service model being implemented. Rather, through analysis of the way GPs discuss the service, the study appropriately seeks to understand GPs perspectives on the proposal. This study does not address the barriers and facilitators to the patients’ engagement with the service, and this topic is an important recommendation for future research.

## Conclusions

Successful implementation of any new model of service delivery in primary care relies on engagement by GPs. This study identified several issues related to engagement with the main negatives being concerns about potential for confusion, incongruence of information and advice, disconnect with other schemes and initiatives, reticence by GPs to embrace the proposed service due to perceptions of loss of control of patient care and lack of belief in need and benefits, resistance to change because of lack of familiarity with the procedures and the personnel, and reluctance to trust in their skills and abilities. GPs also recognised the potential positives of the extra support for their patients, and improved access for remote patients to health professionals with specialist knowledge. The findings can be used to identify possible strategies to improve engagement and uptake of a remotely-delivered ‘care support team’ model by GPs.

## Additional files

Additional file 1: Consolidated criteria for reporting qualitative studies (COREQ): 32-item checklist. Evidence to support the quality of the reporting of the study. (PDF 67 kb)

Additional File 2: Codes within each criterion of the APEASE framework and supporting participant quotations. Additional participant quotations supporting each theme. (PDF 173 kb)

## Abbreviations

APEASE: Affordability, practicability, effectiveness, acceptability, safety/side effects, equity
COM-B: Capability/opportunity/motivation - behaviour
COREQ: Consolidated criteria for reporting qualitative studies
GP: General practitioner
OA: Osteoarthritis
