# Supplementary material for: General practitioners’ perspectives on a proposed new model of service delivery for primary care management of knee osteoarthritis: a qualitative study
Source: BMC Fam Pract. 2017 Sep 7;18:85. doi: 10.1186/s12875-017-0656-7 (PMC5590156; doi:10.1186/s12875-017-0656-7)
Supplement: Supplementary file 1 — Consolidated criteria for reporting qualitative studies (COREQ): 32-item checklist. Evidence to support the quality of the reporting of the study. (PDF 67 kb) [file 12875_2017_656_MOESM1_ESM.pdf]

Additional File 1

Consolidated criteria for reporting qualitative studies (COREQ): 32-item checklist

Adapted from:
Tong A, Sainsbury P, Craig J. Consolidated criteria for reporting qualitative research (COREQ): a 32-item checklist for interviews and focus groups. *International Journal for Quality in Health Care*. 2007. Volume 19, Number 6: pp. 349 – 357

No. Item	Guide questions/description	Comment
**Domain 1: Research team and reflexivity**		
Personal Characteristics		
1. Inter viewer/facilitator	Which author/s conducted the interview or focus group?	Reported in manuscript
2. Credentials	What were the researcher's credentials? E.g. PhD, MD	Reported in manuscript
3. Occupation	What was their occupation at the time of the study?	Reported in manuscript
4. Gender	Was the researcher male or female?	Reported in manuscript
5. Experience and training	What experience or training did the researcher have?	Completed a short course on qualitative research and experience in several previous qualitative studies
Relationship with participants		
6. Relationship established	Was a relationship established prior to study commencement?	Reported in manuscript
7. Participant knowledge of the interviewer	What did the participants know about the researcher? e.g. personal goals, reasons for doing the research	Participants knew that interviewer was undertaking the interviews for the study and who the lead investigators were.
8. Interviewer characteristics	What characteristics were reported about the inter viewer/facilitator? e.g. Bias, assumptions, reasons and interests in the research topic	Reported in manuscript (Interviewer was not involved in development of the model)
**Domain 2: study design**		
Theoretical framework		
9. Methodological orientation and Theory	What methodological orientation was stated to underpin the study? e.g. grounded theory, discourse analysis, ethnography, phenomenology, content analysis	Data analysis was underpinned by a constructionist theoretical

		perspective
Participant selection		
10. Sampling	How were participants selected? e.g. purposive, convenience, consecutive, snowball	Reported in manuscript
11. Method of approach	How were participants approached? e.g. face-to-face, telephone, mail, email	Reported in manuscript
12. Sample size	How many participants were in the study?	Reported in manuscript
13. Non-participation	How many people refused to participate or dropped out? Reasons?	N/a. Since participants approached us in response to advertising of study through networks, non-participation cannot be determined.
Setting		
14. Setting of data collection	Where was the data collected? e.g. home, clinic, workplace	Reported in manuscript
15. Presence of non-participants	Was anyone else present besides the participants and researchers?	N/a
16. Description of sample	What are the important characteristics of the sample? e.g. demographic data, date	Reported in manuscript
Data collection		
17. Interview guide	Were questions, prompts, guides provided by the authors? Was it pilot tested?	Interview guide reported in manuscript. Not pilot tested.
18. Repeat interviews	Were repeat inter views carried out? If yes, how many?	No repeat interviews because too much burden for participants
19. Audio/visual recording	Did the research use audio or visual recording to collect the data?	Reported in manuscript
20. Field notes	Were field notes made during and/or after the interview or focus group?	Reported in manuscript
21. Duration	What was the duration of the inter views or focus group?	Reported in manuscript
22. Data saturation	Was data saturation discussed?	Reported in manuscript
23. Transcripts returned	Were transcripts returned to participants for comment and/or correction?	No return of transcripts because too much burden for participants
**Domain 3: analysis and findings**		
Data analysis		
24. Number of data coders	How many data coders coded the data?	Reported in

		manuscript
25. Description of the coding tree	Did authors provide a description of the coding tree?	Reported in manuscript
26. Derivation of themes	Were themes identified in advance or derived from the data?	Reported in manuscript
27. Software	What software, if applicable, was used to manage the data?	Reported in manuscript
28. Participant checking	Did participants provide feedback on the findings?	No checking of findings by any participants because of burden to participants
Reporting		
29. Quotations presented	Were participant quotations presented to illustrate the themes/findings? Was each quotation identified? e.g. participant number	Reported in manuscript and Additional file 1
30. Data and findings consistent	Was there consistency between the data presented and the findings?	Original data provided. Consistency established by our independent GP
31. Clarity of major themes	Were major themes clearly presented in the findings?	Reported in text and table
32. Clarity of minor themes	Is there a description of diverse cases or discussion of minor themes?	Reported in text and table
