# Supplementary material for: General practitioners’ perspectives on a proposed new model of service delivery for primary care management of knee osteoarthritis: a qualitative study
Source: BMC Fam Pract. 2017 Sep 7;18:85. doi: 10.1186/s12875-017-0656-7 (PMC5590156; doi:10.1186/s12875-017-0656-7)
Supplement: Supplementary file 2 — Codes within each criterion of the APEASE framework and supporting participant quotations. Additional participant quotations supporting each theme. (PDF 173 kb) [file 12875_2017_656_MOESM2_ESM.pdf]

**Additional File 2**

Codes within each criterion of the APEASE framework and supporting participant quotations:

| **Affordability** | **Participant quotes** |
|---|---|
| Cost to patients will affect patient engagement | GP3 *Ideally it should be funded, so provided for free*.<br><br>GP2 *If there's a cost [to patients], that could be a problem.*<br><br>GP11 *But look I guess with any of these kinds of things cost is always an issue. Who's going to fund it?* |
| Cost to patients will affect GP engagement | GP3 *Well, if you say to GPs that by sending them to a 'care support team' people will get more affordable, accessible allied health…* |
| **Practicability** | **Participant quotes** |
| Efficiency of referral procedure | GP7 *It's how much information the team needs, how much of it is not necessary, well will they repeat that themselves by way of that initial assessment. I think it's important not to create more unnecessary work, I mean obviously any referral has to come with a minimum data-set so that you make sure what you're sending is appropriate for what the service is designed to do.*<br><br>GP3 *You know, you can have a system that says if you're going to treat someone for OA a box pops up that says, have you thought of getting a 'care support team'? But you're going to find that's logistically really messy and the IT people are going to tell you it's too hard. And even if you do do it you're not going to be able to get it working on all of the templates in all the key surgeries. But that is a real challenge.*<br><br>GP7 *It depends how simple the [referral] process is. I mean, if you've got a referral template that you're simply filling in a few details, that doesn't take a lot of time*. |
| Efficiency and effectiveness of ongoing communication | GP1 *I think it comes down to the practicalities to be honest for a lot of these systems whether they succeed or fail, and that's about taking time with the communication that was set up and getting the foundation in place to be effective.*<br><br>GP10 *So, we don't have to spend a lot of time sort of when the patient comes back and says that, you know they're having problems or it's not getting better or there's some kind of issue, we're not having to spend a lot of time asking "Well, did you see this person? What did they do, what did they say?" You know, "What sort of prognosis did they give you?" So, if we've got it all there then we can say, "Well, I know she saw you and then this is the plan and she said it's going to take a while but she is confident that you'll get better." If we get that sort of professional opinion from someone else we can sort of help interpret it to the patient because often they come away with a different impression or they've sort of got their expectations wrong and they think they should be better in a week instead of, you know, "Well, this will take a few months." So, I think that's really important that we know from another professional what they think the prognosis is and what they think they can offer and what the plan actually is.*<br><br>GP5 *I guess making sure that the lines of communication between the team and between the GP work really well.*<br><br>GP2 *So as long as I was getting fed back with some information….I'd really appreciate that.*<br><br>GP10 *Communication would be important to us, so we're kept in the loop.*<br><br>GP10 *A timely letter. And you know, with a summary of the assessment and the suggested referrals and you know when the anticipated time that the patient could access those services would be…you know, when the follow-up would be, when we'd need to see them, what else we should do in terms of monitoring as well.*<br><br>GP1 *It's just about that onward communication with them, so you would like to hear about what… you know, were they setting goals, or what contact were they going to have with the patients, and so when they come back you know what to expect….*<br><br>GP6 *So they come back to the see the GP, the GP is not really sure what's going on or where they've been or come with a request* |

| | |
|---|---|
| | *for such and such from this team and doesn't really know why or what or whether it's appropriate.* |
| Fitting in with existing initiatives | GP10 *… and somehow, as I said, tie that into like an existing Team Care arrangement or GP management plan would be really great.*<br><br>GP3 *And they've gone ahead with the medical home model to say people need to be connected to the practice. So there's all these other things that are happening in the background that will influence how GPs engage with a programme like this. And thinking about how this will fit into the regular work of a GP will make a big difference, I think, to whether it succeeds or fails.*<br><br>GP10 *We're familiar with the GP management plan already and it does sound like it's sort of working in a similar way where you know it's multi-disciplinary, it's for a chronic disease, and you know we formulate a Care Plan and communicate with each other about the coordinated care.*<br><br>GP3 *Because the chronic disease management plan often includes four or five chronic diseases and four or five health practitioners, that kind of more holistic view. How will this 'care support team' get a sense of that and link it with a chronic disease management plan? Like, it could get quite messy so that would be the area that's quite complicated.* |
| **Effectiveness** | **Participant quotes** |
| Is there a need? | **Negative**<br><br>GP6 *I'd have to be convinced that that team, for the vast majority of patients, was able to deliver something I wasn't able to do within either my own skill-set or within the practice skill-set.*<br><br>GP6 *I suppose, again my initial feeling is well, I've done the job for like 30 years and one way or the other most patients seem to have done okay with something like OA, and it's just straightforward diagnosis and can be a bit difficult to manage the pain but I suppose we're getting a little bit closer to, a point of view that my 'care support team' would be, you know, my practice nurse or a physio or the dietician, so these are people within the practice that I'm familiar with and so we have a shared record of giving information back.*<br><br>GP3 *But if the 'care support team' just becomes another silo which tells patients what they need but doesn't actually improve access to those additional services then it won't work.*<br><br>GP6 *I suppose my initial thing is that there's a tonne of other patients that I'd love to have a 'care support team' around; I'd much rather have access to or finances for, you know, the help the service provided, um say osteoarthritis doesn't jumped to the top of my tree of clinical issues and problems that I find difficult to manage.*<br><br>GP3 *And that's certainly a risk that you'll face with this intervention of are the people who are just talking over the phone really going to have any more insight or understanding of what the patient needs than the GP?*<br><br>**Positive**<br><br>GP5 *…sort of reinforcing and building on what I'm saying in a one on one consultation I guess it would be better because that way I feel as though I don't need to do it all. And you know sort of that the message isn't living or standing on whatever I'm saying, it is going to be reinforced.*<br><br>GP7 *Yes that's good, because I think with an aging population, we're going to be seeing more of osteoarthritis full stop.*<br><br>GP3 *You know, some kind of stuff that provides hope or a sense of encouragement or that checks that patients actually understand what we're saying.*<br><br>GP6 *I think anything, anything that integrates care is, can only be a good thing.*<br><br>GP2 So *I think that it will be like a one stop referral shop, it will be fantastic as a team behind the patient, and that could be good.*<br><br>GP1 *I think the benefits would be… I think there would be a more… there could be a more holistic view that you could just be part of rather than, you know, just kind of trying to over medicalise it.* |

| | |
|---|---|
| | GP1 *…instead of writing Voltaren and saying use that now and again when it's bad, and take paracetamol, and let's get some exercises, and let's lose some weight and, you know, these are all the things we need to look at, but here's somebody who's actually going to monitor and encourage and be part of all this journey with you.* |
| Will it improve outcomes? | **Negative** |
| | GP8 *And what I found was the patients didn't mind the phone call; they loved it. But they actually didn't lose weight. They didn't reach the goal that we were hoping to achieve.* |
| | GP3 *It's mostly just therapeutic nihilism, isn't it? You know, that we've been giving this advice for years and it doesn't usually work.* |
| | GP3 *Like, if patients say they like it and you can then go back to GPs and say patients have said this is good, you've at least got a chance of getting GPs interested in trying it again. Whereas if it's some other new-fangled thing that is overly complex to use and patients don't like it anyway then you won't get that far.* |
| | GP3 *…to me having an extra team that over the phone are going to repeat some of this advice, how much extra benefit will it make?* |
| | GP9 *I sort of struggle to see how it would be useful in improving the patients motivation even though there are all these people in the background like if they're not actually actively there and telling the patient what they could do or offering solutions it's I don't see how a purely remote set up could be helpful if you get what I mean.* |
| | GP9 *Well I think a mixture of remote and face to face would be good because what would they really get from a remote interaction with a physiotherapist? And I mean if it was a remote psychologist totally see how that could work but yeah not sure how the other allied health set up could actually work remotely.* |
| | GP4 *And also being able to describe, how do you describe an exercise over the phone? You need to see, that's like my chart has got photographs and so on which would be more beneficial than some sort of advice program over the phone.* |
| | GP6 *I'm … not sure how effective patients would find speaking to someone about, you know, exercise or … as opposed to face-to-face and having that personal contact. For some patients it might work fine but … you know, again probably if it was my OA, I would be thinking, "Alright, I'm not sure, I've got an awful lot out of that" but I haven't experienced it, so but it's probably nicer seeing someone face-to-face.* |
| | **Positive** |
| | GP11 *But certainly that extra interaction, that extra accountability, that extra bouncing off somebody to do the change maybe more than they would obviously get by coming back to their GP who's probably only going to see them every two three months at the most. So all of that extra contact should you would hypothesise flow into more motivation and more actual change.* |
| | GP7 *I think in terms of motivation and encouragement, to have care support who are there for other reasons, that if they then have an opportunity to prompt, encourage, reward and say, "Well done," I think that can only be a good thing.* |
| | GP4 *So maybe the dietician and the team would be more successful than we would be in terms of promoting a diet that they'll accept…* |
| | GP3 *I guess if it's much more specific advice of more nuance on parts of exercise and weight loss that I as a GP don't know about well maybe that will help.* |

| **Acceptability** | **Participant quotes** |
|---|---|
| Trust | GP6 *I'd have to be convinced that the team, for the vast majority of patients, was able to deliver something I wasn't able to do within either my own skill-set or within the practice skill-set* |
| | GP3 *And to assume that the quality of their communication skills will be any better or worse than GPs I guess is the concern.* |

| | |
|---|---|
| | GP7 *What you would need to know is that they're competent to do what's being asked of them, you know, in terms of training. That they in turn have got access to resources and that they know when to refer back. The checks and balances. Because I would not want to think that they would be doing anything that would be exacerbating the problem or creating other difficulties, such as soft-tissue injuries, because of inappropriate management. Or that they didn't know that if there isn't an improvement after x-length of time, or there's no response to that particular treatment modality that's been outlined, that they would know to refer back for review.* |
| | GP3 And, you know, GPs are quite sophisticated communicators. People often don't realise it but we do realise it when we send people off to other interventions particularly telephone based interventions. |
| | GP3 I do have a bit of concern if I don't know who they're going to get. Like, I think your thing said there could be pharmacists, psychologists, physios. And each of those groups have very different expertise so how are you going to decide which type of expertise the individual patient needs. Like, just assuming that there's generic person that can do all of it, I think you'll find it's very much dependant on the type of people you get to deliver the intervention. It won't be what their clinical discipline is, it will be on their communication skills and their ability to engage. |
| | GP3 But it tends to get people's backs up because they kind of have this us and them tension. So the ability is getting the right person providing the care support, the person who's actually got the right communication skills and right insight and right expertise. |
| | GP3 Whereas I could say to them, look, if you go through this 'care support team' you're going to actually be sure of accessing people who are really shit hot with osteoarthritis. |
| | GP8 I think if patients came back and said, "That was a really good service. Really worthwhile" and we got the clinical outcome that would be the thing that would encourage me to keep it going. |
| | GP7 But I think, and I think that's the other thing that if you can say to GP's, "Look, this works because we can show these following improvements, you know, the following improvement," then that may well make them more enthusiastic to engage with it. |
| | GP10 If these people - I guess if all the Allied Health have knee OA or OA as a sort of special interest, that helps as well because I mean like we're sending them - you know, sending the patients to the right people. |
| Information security and confidentiality (Trust) | GP10 I guess, you know, we'd have to know how the information would be managed and we'd have to be clear about who is responsible |
| | GP8 And allowing patient confidentiality to go up in the Cloud and having external people looking at the confidential information; that really concerns me. |
| Familiarity with the service (Trust) | GP11 … what actually is being proposed by what … who's seeing them? How many visits? What are they covering? What's the purpose?) |
| | GP1 *I think it's just what I said that I think for me, and I think probably for most practitioners not just GPs, but I think the relationship matters to know what they're actually going to be able to be offering so that you can actually speak of it as something you know rather than just something you've been told this is for OA and here's your referral.* |
| | GP10 *I guess I wanted to know what the overlaps were with team care arrangements, so how that would be different to a team care arrangement and how that would be funded and how easily patients can access that and how long that would go for as well.* |
| | GP10 *We'd have to be clear about who is responsible - you know what the role of a GP is in this process and what the role of the 'care support team' is in the process as well.* |
| | GP 11 *…as long as each team member you're dealing with has a reasonably similar they're on the same page.* |
| | GP2 *If you have a brochure or a pamphlet outlining all the benefits of it* |

| | |
|---|---|
| Personal relationships | GP6 *My 'care support team' would be, you know, my practice nurse or a physio or the dietician, so these are people within the practice that I'm familiar with and so we have a shared record of giving information back. The idea of handing a patient over to, you know, an anonymous group of people, I find I don't see a great attraction.*<br><br>GP7 *Our experience of working with other groups is that the closer you work, the better the relationships, the more you understand each other's roles and responsibility… So, if you're on questioning terms this means, let's say I phone up for a bit of advice or if you've got particular concerns about a client they're already seeing, to me it frees up that movement of patients into the service and hopefully back again. The care support [team] know the practice and know the doctors that work in that practice and can let them back more confidently than they might do otherwise.*<br><br>GP1 *It's being new, if the 'care support team' came and met us….If the 'care support team' were people that we'd actually met and we knew what they were capable of and they could tell us, well, you know, these are the sorts of things we can do and offer, that they become names to us, you know, rather than just an anonymous, you know?*<br><br>GP10 *And another thing that's really helpful is just to have the one key person, kind of like your case manager, that sort of concept that you know the GP can - like a liaison person for that particular patient so we can go to that person if we've got any questions or concerns instead of sort of wondering who to contact.* |
| Burden of care on GPs | GP9 I think I mean for me to know that there's a team that's willing to support my patient with osteoarthritis would be a huge help because right now I'm sort of I'll have to find a physio where the patient lives and a dietician where the patient lives and potentially a specialist and a phycologist and they're all separate on separate days separate areas so yeah it would be really nice to have everyone in one place I guess. And or have a team that work together and do this all the time.<br><br>GP4 That may mean that some of my responsibilities are less.<br><br>GP7 If what's being proposed actually takes some of the load away from frontline GP's that might well be the incentive that you need. You know, there's something in it for them, not just the patient.<br><br>GP10 I guess if it made referring easier for us then that would be another, I guess, enabler. So, in terms of, "Okay. Well, I should…" if I'm thinking I have to refer to a dietician and then an exercise physiologist and find somewhere for the patient to get a bit of physio, and then they might want to be a bit of Tai Chi or...so there's quite a few sort of services that we'd have to refer to. So, if your 'care support team' could help with some of that, that would just make, I guess, the whole process a lot easier for us in terms of managing OA.<br><br>GP10 And that would be helpful for the GP instead of sort of trying to figure out, you know, "Where can I send these people?" The 'care support team' could take care of that.<br><br>GP11 So whilst I'm not trying to abdicate responsibility for GPs to do this stuff it may be that if you're working in a team there are other people who are doing it and so the actual overall thing does happen but it's just someone else doing it. |
| Perceived sustainability (Trust) | GP3 *And that's why you sometimes get this kind of pessimism from GPs. It's not that they don't want better interventions, it's just that they're sceptical that they will truly become a routine easily accessible part of practice.*<br><br>GP3 *And they won't be there forever and they're not going to be like, suddenly incorporated into general practice forever.* |
| Financial incentivisation (not used in manuscript) | GP5 GPs aren't in it for the money but it does help. So you know if you've got something that you go OK this will benefit my patient and I might get paid a little bit more for it, you go OK, I don't really have anything to lose here.<br><br>GP10 I think incentives never sort of go astray. I don't think there, you know, necessarily needs to be, but I think to sort of make GPs remember it, an incentive could help. [A monetary incentive?] Yeah. Yeah. |

| **Side effects / safety** | **Participant quotes** |
|---|---|
| Worsening of outcomes | GP3 *Because this 'care support team' basically just says, it sounds like you need help with exercise, I'm going to refer you to a physio. The patient will probably say, my GP already suggested a physio and I just haven't followed through. And you could end up just putting an extra silo of care without actually doing anything more than…* |

GP6 *And you know, it's a potential breakdown of that continuity of care, I mean, how does the patient access it? How do we exchange information? You know, how do I as the GP, how do I keep a sense of being involved in an ongoing situation? Am I just handing over to, you know, a group of other people and I wash my hands of them?*

GP8 *So, anything where we're going to be, you know, making more time for us, we were uploading information and transferring information, all that I would be discouraging.*

GP2 *I suppose depends how it's fed back. Sometimes you get a report, which is great, sometimes they pick up all these ... you know, some ... they'll pick up a whole lot of problems, which create more problems for you and the patient, which weren't ever there to start with…*

GP2 *…from a GP point of view I'd be concerned it might be conflicting referral advice being given.*

GP6*…maybe difficulties with the breakdown in communication, again - so they come back to the see the GP, the GP is not really sure what's going on or where they've been or come with a request for such a such from this team and doesn't really know why or what or whether it's appropriate. So, that loss of continuity in GP involvement could be not ideal.*

GP3 *....and particularly with the issue of how will it integrate with chronic disease management plans. Like, if this ends up with the GP refers to the 'care support team' and the 'care support team' says you need to see a physio, go back to your GP and get a chronic disease management plan, my idea would have been well, you should have done that in the first place and why can't the 'care support team' then just help do the chronic disease management plan like, fill it in. Like, it could get quite messy so that would be the area that's quite complicated.*

GP2 ... *So the first we hear about it is after they've already asked the patient, the patient's already part of it....And I'm sort of thinking, well what's all that about, you know, and I sort of ... I just file it and leave it there and don't think much about it, so I suppose my concern would be the fragment health care. I think the GP is really well placed to coordinate all aspects of the patient's care. So, as long as it's not all fragmenting care, it would be good.*

GP6 *…how does your GP remain part of that team?*

GP6 *Again; that loss of ownership that you're, you know, a lot of general practice is about looking after and caring for the patient.*

GP2 *Would you, being a control freak, would you like ... if someone in the 'care support team', be saying, oh, if you need surgery, I'd recommend you see this surgeon, who ... someone I wouldn't use, so -… [So losing control?]…Yeah.*

GP 1 *So I think you should continue to be involved but hopefully in a kind of a… what would you call it, you know, in a triangular way that the patient would see that you have contact with the 'care support team', and so would you, but you would actually have a big overlap with all three of you really.*

GP6 *So, that loss of continuity in GP involvement would be not ideal.*

GP3 *And to assume that the quality of their communication skills will be any better or worse than GPs I guess is the concern. Like, will they be saying the same thing as the GPs, will they be adding to the complexities, will they be giving advice that might be inappropriate*

GP5 *…there's a possibility that the patient won't gel with them or that the way that they approach the problem is going to be a little bit different to mine… every now and then it's some seemingly innocent or innocuous comment the patient turns over and then brings it back to you and you have to sort of spend time addressing that.*

GP2 *I'd be interested to know the 'care support team' were saying what I was saying, especially if I was trying to sort of get an exercise physiologist, what they'd be saying about that, so I suppose that from a GP point of view I'd be concerned it might be conflicting referral advice being given*

GP3 *But that must be confusing for the punter because they're bombarded so much with different opinions and different information ...And there was so many different subtle approaches that the health literate consumer might actually get overinvolved when really all they should be doing is losing weight and exercising. They're often looking at other things.*

| | |
|---|---|
| | (GP3) *Like, will they be saying the same thing as the GPs, will they be adding to the complexities, will they be giving advice that might be inappropriate?* |

| **Equity** | **Participant quotes** |
|---|---|
| Patients need individualised care (Patient diversity) | GP3 *There is a risk that people go, well, they were just following a script, they weren't tailoring it to me, they didn't understand me, they just told me stuff that I could have read in a book anyway.*<br><br>GP2 *Some patients might have very mild arthritis and they don't want to [access the service], some patients are just waiting for their new knees, that won't be relevant, or they might not feel it's relevant.*<br><br>GP3 *I'm thinking that a one size fits all [service] that will work because someone has OA of the knee is a little bit of a pipedream.*<br><br>GP4 *Of course they've got to have good hearing, sometimes they struggle to understand as they get older exactly what's being conveyed to them.*<br><br>GP3 *Because even if they do help a bit they won't help all patients.*<br><br>GP5 *I guess obviously if there was any possibility of talking to someone with you know a bit of local knowledge for a particular geographic area that's advantageous.* |
| Acceptability to patients (Patient diversity) | GP1 *I think the negatives is that the patient may well feel oh right, you know, they have been sort of sidelined or kicked off to something that, you know, they're not being understood because it's actually a fairly major impact on their day-to-day living.*<br><br>GP8 *And what I found was the patients didn't mind the phone call; they loved it.*<br><br>GP4 *The team would have to ring them on occasions to see how they're going as I would think the patient themself might become lazy in terms of making the phone call.*<br><br>GP4 *Of course they've got to have good hearing, sometimes they struggle to understand as they get older exactly what's being conveyed to them.*<br><br>GP5 *Probably the down side would be that there is going to be a group both of patients and GPs who just don't want to engage with that type of model. But I think that will be the case no matter what model is designed or developed.*<br><br>GP1 *I think it's just all sort of like those cold calls, or, you know, people go oh, heck, well what would they know about it, you know, really, what gives the validity that this person can help me.*<br><br>GP2 *Some patients don't like ... they feel it's intrusive, they don't feel ... they don't even want a health assessment from the nurse, they don't want other people ringing them and being involved, so it's a privacy issue on some patients.*<br><br>GP2 *…you've got to at least sell it to the patient.* |
| Access to support and services for rural patients (Patient diversity) | GP9 *I think accessibility is a huge pro, so if it's remote then it can be accessed by phone or internet or something whenever the patient is free.*<br><br>GP5 *And you know there'd certainly be a group of patients who I think need that sort of someone who they feel they can have that constant contact with because particularly in rural areas sometimes actually getting an appointment with your GP is really, really difficult.* |
